# Death and Science: The Existential Underpinnings of Belief in Intelligent Design and Discomfort with Evolution

**DOI:** 10.1371/journal.pone.0017349

**Published:** 2011-03-30

**Authors:** Jessica L. Tracy, Joshua Hart, Jason P. Martens

**Affiliations:** 1 Department of Psychology, University of British Columbia, Vancouver, British Columbia, Canada; 2 Department of Psychology, Union College, Schenectady, New York, United States of America; The Centre for Research and Technology, Hellas, Greece

## Abstract

The present research examined the psychological motives underlying widespread support for intelligent design theory (IDT), a purportedly scientific theory that lacks any scientific evidence; and antagonism toward evolutionary theory (ET), a theory supported by a large body of scientific evidence. We tested whether these attitudes are influenced by IDT's provision of an explanation of life's origins that better addresses existential concerns than ET. In four studies, existential threat (induced via reminders of participants' own mortality) increased acceptance of IDT and/or rejection of ET, regardless of participants' religion, religiosity, educational background, or preexisting attitude toward evolution. Effects were reversed by teaching participants that naturalism can be a source of existential meaning (Study 4), and among natural-science students for whom ET may already provide existential meaning (Study 5). These reversals suggest that the effect of heightened mortality awareness on attitudes toward ET and IDT is due to a desire to find greater meaning and purpose in science when existential threats are activated.

## Introduction

Despite overwhelming evidence for Darwin's theory of evolution (ET) and scientific consensus that intelligent design theory (IDT) is inherently unscientific [1], IDT has received considerable support from the general public, educators, and elected officials [2], [3]. Many schools include IDT in science curricula; 25% of U.S. high-school biology teachers devote at least some class time to the topic, and nearly half of those view IDT as a “valid scientific alternative to Darwinian explanations for the origin of species” [4]. Although a Dover, PA, court ruled in 2005 that schools could not include IDT in Pennsylvania science curricula, the debate is far from over. In 2008, Louisiana passed a bill permitting science teachers to use outside sources—including those supporting IDT—in curricula, and in 2009 the Texas state education board voted to allow IDT to be taught alongside ET in science classes.

This debate is not restricted to the U.S.; in 2006 the Social Sciences and Humanities Research Council of Canada (a major branch of the nation's federal research funding agency) refused to fund research examining the (presumably negative) effects of IDT's notoriety, on the grounds that there was not “adequate justification for the assumption …that the theory of Evolution, and not Intelligent Design, was correct” [5]. In 2009 the province of Alberta passed a law that may allow parents to remove children from courses covering evolution [6]. Given this international climate of continuing support for IDT and doubt about ET, despite IDT's lack of scientific credibility and the large body of scientific evidence supporting ET, it is likely that psychological motives, beyond logic and reasoning, underlie the willingness of educated individuals such as teachers and school board members to question ET and accept IDT as a viable alternative.

Indeed, psychological motives, which often operate implicitly, can shape sociopolitical beliefs and ideologies. A comprehensive meta-analysis found that political conservatism is at least partly rooted in the basic need to manage feelings of threat and uncertainty [7]. Specifically, conservative attitudes relate positively to death anxiety, intolerance of ambiguity, and low self-esteem. Other research shows that increasing existential anxiety by reminding people of their own mortality influences attitudes toward hypothetical political candidates [8], actual political figures, and foreign-policy strategies [9], [10]. Thus, although dispositional political and religious ideologies may be central factors underlying the success of the IDT movement and corresponding doubt about ET, fundamental psychological motives, such as the need to maintain psychological security, are also likely to influence these beliefs when activated. (Cognitive processes also play a role in shaping these views; studies have shown that young children, and adults with Alzheimer's who cannot remember learned knowledge about the origins of objects, tend to show a preference for teleological and other essentialist explanations for the origins of objects and organic phenomena [11], [12].).

In the present research, we examined whether implicit concerns stemming from individuals' awareness of their own mortality might be a cause of the widespread support for IDT and corresponding skepticism of ET seen among a wide range of individuals in North America. We tested the hypothesis that heightened mortality awareness would lead individuals to embrace IDT and reject ET; in other words, that shifting one's opinion on these theories is a “terror management” strategy—stimulated by the basic need to maintain psychological security [13].

### Terror Management and Acceptance of Intelligent Design versus Evolution

According to terror management theory (TMT) and findings, humans' awareness of their mortality has the potential to produce debilitating anxiety, so individuals tend to respond to life's frequent mortality reminders by employing psychological mechanisms that inhibit death-related thoughts [13]. These include enthusiastic adherence to meaningful conceptions of reality (i.e., “worldviews”), such as religious and political belief systems. Worldviews may promote a sense of immortality—buffering existential anxiety—by construing the universe as an orderly, comprehensible, predictable, and meaningful place where death can be literally or symbolically transcended. For example, a sense of literal immortality may be provided by religious belief in an afterlife [14], [15], and a sense of symbolic immortality may be provided by “living on” through one's accomplishments, offspring, or cultural affiliations [16].

IDT may be one such equanimity-providing worldview, albeit a slightly unusual one. IDT proposes that naturalistic accounts are insufficient to explain complex organic phenomena and that therefore an intelligent and presumably supernatural “designer” is responsible for the origin of all life [17]. IDT may calm existential concerns through the implications of its assertion that human life was intentionally created, rather than resulting from seemingly random and meaningless forces of nature (i.e., natural selection). This may allow for symbolic immortality—taking comfort in something larger and more significant than one's own brief life—via the understanding there is a purpose to the human enterprise.

Furthermore, whereas many mollifying religious and ideological worldviews have little appearance of being evidence-based or rationally derived, IDT is presented as a scientific theory, and was proposed and developed by scientists at major academic institutions [17], [18]. This may make IDT existentially appealing in a broader way than most worldviews, which tend to be adhered to in response to existential threat only by dispositional devotees [19]. By couching their theory in explicitly scientific terms, IDT's authors have made the theory amenable to educated individuals with some level of basic scientific knowledge, who may be hesitant to adopt explicitly religious resolutions to existential concerns. For the average educated American, it may be difficult to embrace a Biblical view of the world and simultaneously maintain a feeling of belongingness in the broader culture of Western science-educated individuals. Because IDT superficially appears consistent with both the scientific and religious worldviews, a wide range of individuals (e.g., science teachers, university students, religious believers) may feel they can support IDT and maintain allegiance to their science-educated and/or religious communities. (Some *literal* immortality may be inferred from IDT as well, based on the assumption—never directly stated by the theory's proponents—that a universe with a supernatural creator might allow for life after death.).

In contrast, on its face, ET does not confer any sense of greater meaning or purpose, instead asserting that human life is the result of the same natural forces that produce viruses and cockroaches. Although scientists may find meaning and purpose from the notion that all life is connected by virtue of resulting from the same explicable biological forces, for the average non-scientist ET may seem existentially bleak. Evolutionary biologist Richard Dawkins noted such responses among readers of his books on evolution: “A foreign publisher of my first book confessed that he could not sleep for three nights after reading it, so troubled was he by what he saw as its cold, bleak message. Others have asked me how I can bear to get up in the mornings. A teacher from a distant country wrote to me reproachfully that a pupil had come to him in tears after reading the same book, because it had persuaded her that life was empty and purposeless. He advised her not to show the book to any of her friends, for fear of contaminating them with the same nihilistic pessimism” [20].) Consequently, existential concerns may lead many individuals to question ET, particularly if IDT is an available option. Indeed, experimental reminders of one's mortality, known to induce a state of “mortality salience” (MS), have been found to reduce liking of essays which, consistent with ET, emphasize humans' animal nature. Similarly, priming individuals with reminders of their biological similarity to animals increases death-thought accessibility, as does reading about ET, among Creationists [21]–[23]. More broadly, the stronger individuals' belief in evolution, the less likely they are to believe in the “soul” or afterlife, suggesting that acceptance of ET may be untenable for those who have taken a more spiritual approach to finding meaning in life, and vice-versa [24]. Thus, ET may be a conceptual obstacle to a search for greater meaning in life, so rejecting or denying ET's veracity may be a means of regulating existential anxiety.

However, based on TMT, existential anxieties might also be expected to promote acceptance of ET in certain individuals—those who are already particularly well versed in the theory. Mortality salience (MS) typically motivates more fervent support of accepted worldviews [13], so science-educated individuals, and natural-science students in particular, might respond to MS by staunchly supporting ET, given that, in the Western scientific worldview, ET is the most widely and empirically accepted explanation for the diversity of life on Earth, and the origin of the human species. At the very least, science-educated individuals—such as the undergraduate psychology students who constitute most psychology research samples—may find themselves unable to reject ET as a way of assuaging existential anxiety, given the importance of ET to their psychology-student worldview. These individuals may nonetheless embrace IDT in such circumstances, given the theory's scientific veneer, but they may fail to see its logical incompatibility with ET—an incompatibility that is at times downplayed by IDT proponents [25]—and avoid shifting their views on the more standard scientific doctrine. Thus, while we expected most individuals to respond to MS by espousing a stronger belief in IDT and weaker belief in ET, we expected university-educated psychology students to respond by embracing IDT, but not necessarily changing their views of ET.

### The Present Research

In five studies we manipulated mortality salience (MS), then presented participants with a passage arguing for ET and/or a passage arguing for IDT, then assessed their views toward the author of each passage and the corresponding theory. Study 1 used a sample of psychology-student undergraduates at largely liberal universities. Given the importance of evolution to psychology students' worldview, we did not expect them to substantially shift their views of ET, but rather to respond to MS by demonstrating greater acceptance of IDT. Study 2 used a more diverse sample of students from across North America; here, we expected increased acceptance of IDT *and* decreased acceptance of ET in response to MS (i.e., an interaction between experimental condition and views toward each theory). We had the same predictions for Study 3, which sampled adult Americans representing a wide range of socioeconomic and educational backgrounds.

Study 4 again sampled psychology students, but additionally manipulated whether naturalism (the scientific perspective underlying ET, but not IDT) was depicted as a source of existential meaning and purpose. This allowed us to examine whether the motive to embrace a scientific theory that provides a sense of greater human purpose is the causal mechanism underlying belief in IDT and/or aversion toward ET in response to existential threat. We predicted that this meaning-in-naturalism manipulation would moderate the effect of MS on views of IDT and ET, such that participants who read the passage depicting naturalism as a source of meaning would *not* respond to MS by espousing greater support for IDT and/or weaker support for ET. Our focus, here, on the importance of seeking greater meaning and purpose in human life as a way of coping with existential dread, is consistent with research demonstrating humans' basic need to maintain a sense of meaning [26]. However, this is one of the first studies to manipulate the meaningfulness of a potential terror-management mechanism (but see [27]), to test whether this fundamental motivation accounts for effects. Study 5 addressed the same issue in a different way; participants were university-level natural-science students, for whom naturalism is already a source of greater meaning. Given these participants' belief system, we expected them to respond to MS with greater support for ET, the theory that provides them with meaning and identity, and greater antagonism toward IDT, which they should recognize as scientifically invalid and inconsistent with their central worldview.

In all studies, we tested whether effects were due to Christianity or other religious beliefs. It has been assumed that public support for IDT results from a Christian desire to reinstate Creationism [28]. Furthermore, studies have shown that MS promotes increased belief in supernatural beings and the afterlife among religious individuals [15], [29], and that religiosity protects against existential threat [14]. However, IDT is not explicitly religious, makes no promise of an afterlife, and reads more like a scientific theory than a religious one. Thus, IDT may provide existential benefits without heightening religious belief, and it may do so even in non-religious individuals—which would have major implications for the scientific views of individuals who either do not have strong religious convictions or would like to reconcile their religious beliefs with their science education.

### Ethics statement

For all studies in the present research, behavioral research ethics board approval was obtained from the University of British Columbia or Union College, and all subjects provided written informed consent (for participants who completed the study via the internet, consent was provided by clicking a designated button on-line; this was approved by the UBC Behavioral Research Ethics Board).

## Methods

### Study 1

122 undergraduate psychology students (72% women) at Union College (*n* = 53) or the University of British Columbia (*n* = 69) were randomly assigned to write about the thoughts and feelings aroused by imagining either their own death (MS condition) or dental pain, a typical control manipulation used in TMT research to ensure that effects attributed to MS are not in fact due to general negative affect or arousal [13]. Participants then completed the Positive and Negative Affect Schedule (PANAS; [30]), before reading a passage arguing for ET, “written by Professor Richard Dawkins, a famous evolutionary theorist,” and a passage arguing for IDT, “written by Professor Michael Behe, a famous scientist who argues for the theory of intelligent design.” These 174-word similarly styled passages were excerpted from the authors [17], [18], [31] (see Text S1). Neither referred to religion or belief; instead, both read as descriptions of, and empirical support for, a scientific theory. Thus, if participants responded to MS by accepting IDT, this could not be attributed to a desire to embrace religion per se.

Each passage was followed by a 6-item scale assessing participants' views about the author's expertise and their belief in the theory referred to in the passage (based on [32]). Specifically, participants rated each author, using a 9-point scale, on intelligence, knowledge, agreement with his views, and truth of his opinion. They then rated their agreement with two statements, on a 5-point scale: “Evolutionary [Intelligent design] theory is a solid theory supported by a great deal of evidence” and “Evolutionary [Intelligent design] theory is the best explanation we have of life's origins.” (It is noteworthy that although evolutionary theory addresses questions about the origin of life for each species, not the origin of life from non-life, it is very commonly presented in this way, and, in fact, the term “origin” can connote either ancestry or inception of life. However, in case this wording might have affected results, we re-analyzed the main effects and interactions in all studies excluding this item, and found that all effects held when scales were based on the remaining 5 items; one minor exception was in Study 5, where the interaction was significant only at the one-tailed level, *p* = .05, and the main effect of MS on Behe-IDT was no longer significant, *p* = .12. Interested readers should contact the authors for more information on these subsidiary analyses.)

The resulting 6-item scales, computed using standard scores were reliable based on Cronbach's *α*s; these were .84 for Behe-IDT and .85 for Dawkins-ET. The scaling of these 12 items was also supported by a varimax-rotated factor analysis, showing that all item loadings ranged from .62–.82 on predicted primary factors, and below .32 on secondary factors. A scree test also suggested a two-factor solution (eigenvalues for the first five components were 4.75, 3.05, 1.23, 0.68, and 0.49). When the factors were allowed to correlate, using a direct oblimin rotation, they were found to be independent, *r* = .00.) Because standard scores were used (here, and in all five studies), means on the two scales (Dawkins-ET and Behe-IDT) cannot be directly compared to each other (both are 0 when standard scores are used). However, to ensure that actual mean responses on these scales were not at floor or ceiling, we also computed scores for each participant by summing across the 6 items on each scale. In no study was the overall mean of these summed scores near ceiling or floor; see Text S2 for greater detail. Order of the passages and scales was counterbalanced; no order effects emerged. It is noteworthy that 4 of these items ask about views of the two authors, rather than directly assessing views of the relevant theories. Although views of the authors are likely to reflect and influence views of their respective theories [33], we wanted to ensure that results are not due to an effect of mortality salience on attitudes toward these two authors but not the theories, so we also ran all analyses using 2-item scales comprising only the last two items, which asked about views toward the theories but not the authors. In all studies, we separately report results for these 2-item scales, after results for the full 6-item scales.

Participants then rated their religiosity on a 10-point scale ranging from 1 (“not at all”) to 10 (“extremely”), completed a measure of intrinsic (Cronbach's α = .72) and extrinsic (Cronbach's α = .79) religiosity [34], and reported the following religious affiliations: Buddhist (13%), Catholic (22%), Christian/Christian Orthodox (15%), Hindu (8%), Jewish (2%), Muslim (3%), Protestant (8%), Spiritual (10%), and none of these (19%).

### Study 2

352 undergraduates (40% women) from 179 universities in 45 U.S. states or Canadian provinces were recruited through an online survey research company (66%; *n* = 232) or in a class at the University of British Columbia (24%; *n* = 83) or Union College (10%; *n* = 37). They followed the same procedure as in Study 1, except that they did not complete the measure of intrinsic/extrinsic religiosity, given an absence of meaningful differences between these measures and the single-item religiosity measure (which was included here) in Study 1. Order of the passages and scales was again counterbalanced; no order effects emerged. Scale scores for Dawkins-ET and Behe-IDT, based on standard scores, were reliable; Cronbach's *α*s = .89 and .91, respectively. Participants reported the following religious affiliations: Buddhist (3%), Catholic (22%), Christian (25%), Christian Orthodox (2%), Hindu (1%), Jewish (4%), Muslim (2%), Protestant (7%), Sikh (1%), Spiritual (11%), none of these (21%), and Other (1%).

### Study 3

832 individuals (55% women), ranging in age from 18–75 years (*Median* = 37), living in the U.S., were recruited through an online survey research company. They followed the same procedure as in Study 2, except that they viewed only one excerpt (arguing for either IDT or ET, as described below), and reported education level, income bracket, social class, and field of work. Religious affiliations were as follows: Buddhist (1%) Catholic (20%), Christian (32%), Christian Orthodox (1%), Hindu (1%), Jewish (3%), Protestant (14%), Spiritual (10%), none of these (12%), and Other (6%). Participants were also diverse in education and socioeconomic status: 3% reported attending “some high school,” 23% had only a high-school diploma, 33% attended “some college,” 32% had a college degree, and 9% had a post-graduate degree; 24% identified as “working class,” 19% “lower-middle class,” 44% “middle class,” 12% “upper-middle class,” and less than 1% “upper class.” Consistent with these ratings, 12% reported an annual income of under $20,000, 25% of $20,001–40,000, 20% of $40,001–60,000, 11% of $60,001–80,000, 8% of $80,001–100,000, and 9% of over $100,000 (15% did not report income).

To control for the possibility of stylistic differences in the excerpts describing IDT and ET influencing results, in Study 3 we changed the excerpts used in Studies 1 and 2, so that the two passages were made to be identical except that one referred to IDT and the other to ET. We did this by combining statements from each of the two passages, actually written by Dawkins and Behe; in the IDT condition, Dawkins' statements were changed to refer to IDT (and presented to participants as if written by Behe), and, in the ET condition, Behe's statements were changed to refer to ET (and presented to participants as if written by Dawkins); see Text S3. Participants were told that passages were written by “Professor Dawkins, a famous evolutionary theorist” (ET condition), or “Professor Behe, a famous scientist who argues for intelligent design” (IDT condition). These sentences were followed by a definition of evolution, as “the natural process of change in inherited traits from generation to generation by mutation, natural selection, and genetic drift” (ET condition), or intelligent design, as “the belief that physical and biological systems observed in the universe result from purposeful design by an intelligent being rather than from chance or undirected natural processes” (IDT condition). These definitions were added to ensure that participants were at least familiar with the basic idea behind the theory they were reading about. Author-theory was manipulated between subjects, because the artificiality of the almost-identical passages would be obvious to participants if they read both. We again computed an author-theory scale using standard scores, which represented participants' agreement with Dawkins and belief in ET (ET condition; Cronbach's *α* = .95) or agreement with Behe and belief in IDT (IDT condition; Cronbach's *α* = .93).

### Study 4

269 UBC psychology students (77% women) followed the same procedure as Study 2, except that after receiving the MS or control induction, half viewed a passage excerpted from Sagan ([35]; see Text S4). They were instructed to “read the paragraph below, written by Dr. Carl Sagan, one of the world's most famous scientists.” They were told that they would later be quizzed on their understanding of the paragraph, to ensure they read the passage carefully and thought about its meaning. All participants then completed the PANAS, then read either the Behe-IDT or the Dawkins-ET passage used in Studies 1 and 2, followed by the same assessment items (here all measured with a 7-point response scale; Cronbach's *α*s, based on standard scores, were .86, Dawkins-ET, and .86, Behe-IDT).

A subset of 104 participants (80% women), roughly equally distributed across the 8 cells, participated in a follow-up study 4–6 months later, in which they completed a measure of evolution acceptance ([24]; Cronbach's α = .85), our single-item religiosity measure, intrinsic and extrinsic religiosity (Cronbach's αs = .80 and .82), and a measure of religious fundamentalism ([36]; Cronbach's α = .85). Intrinsic religiosity was scored omitting 2 items with negative item-total correlations, which, when included, lowered the Cronbach's *α* to .51. No results changed using the full scale instead. These follow-up data were collected because we expected that participants' responses 4–6 months after the experiment could not be attributed to any effects of the experiment, so by testing whether these scores moderate effects, we can further probe whether shifting views toward ET/IDT in response to MS is a strategy utilized only by individuals with certain religious or evolution beliefs.

### Study 5

99 UBC undergraduate and graduate students (50% women) were recruited from natural-science courses and the natural-science library to follow the same procedure as in Study 2. Participants reported the number of university-level biology, physics, and chemistry courses they had taken; range = 2–55, *Median* = 11. Order of passages and scales was counterbalanced; no order effects emerged. Standardized scale scores were reliable; Cronbach's *α*s = .88 (Dawkins-ET) and .90 (Behe-IDT). Reported religious affiliations were: Buddhist (3%), Catholic (22%), Christian (16%), Christian Orthodox (2%), Hindu (2%), Jewish (5%), Protestant (6%), Spiritual (8%), none of these (17%), and Other (3%).

## Results and Discussion

### Study 1

**Figure 1:**
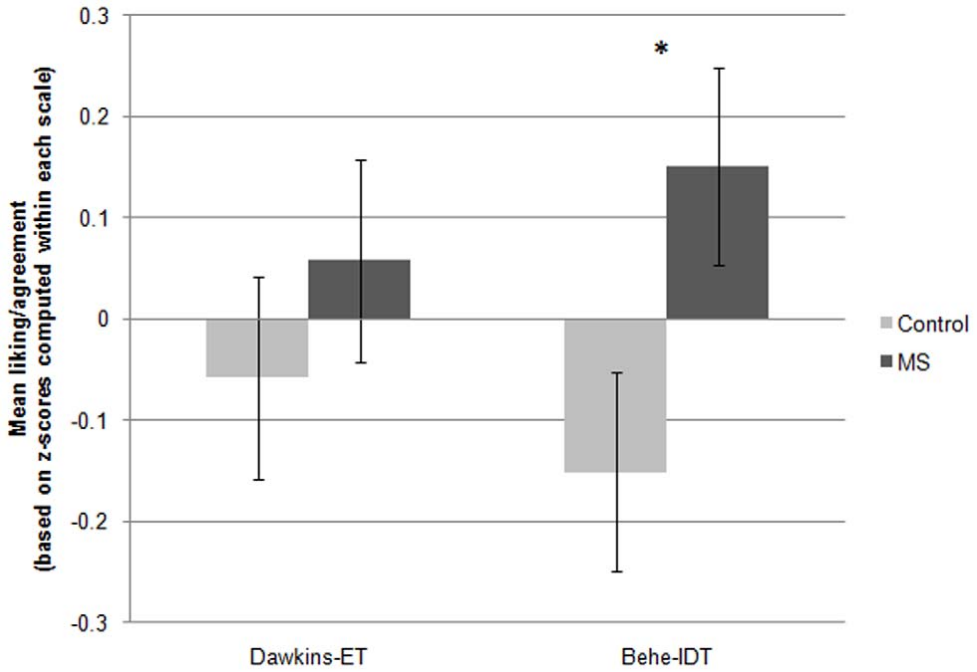
Effects of mortality salience (MS) on liking of Behe and belief in intelligent design theory (IDT), and liking of Dawkins and belief in evolutionary theory (ET), Study 1. **Note.** Values are based on standard scores; means for each scale were computed by standardizing each of the six author-theory items that the scale comprised, around their common mean, and taking the mean of the resulting z-scores. Because the two scales were centered around different means, values on the two scales (Dawkins-ET and Behe-IDT) cannot be directly compared to each other. Based on a *t*-test, the difference between the control and MS conditions on Behe-IDT was significant, *p*<.05. Error bars represent the standard error of the mean. *p<.05.

A mixed-measures analysis of variance (ANOVA) showed no interaction between the within-subjects factor of author-theory (Behe-IDT vs. Dawkins-ET) and the between-subjects MS manipulation, *F*(1,118) = .72, *ns*, indicating that MS did not promote different responses toward the two theories and authors. However, a predicted main effect of MS emerged on the Behe-IDT scale, indicating that MS led to more positive views of Behe and IDT compared to control, *t*(119) = 2.18, Cohen's *d* = .40, *p*<.05 (see Figure 1). This effect held when the two-item scale based on views of IDT was used instead, *t*(119) = 1.72, *p*<.05 one-tailed. No difference emerged for the Dawkins-ET scale.

Religiosity was related positively to Behe-IDT, *r* = .25, and negatively to Dawkins-ET, *r* = −.30, both *p*s<.05, suggesting that more religious individuals tend to support IDT and dislike ET. Intrinsic and extrinsic religiosity showed a similar pattern; *r*s = .28 and −.34 (intrinsic), and .20, −.17 (*p* = .07; extrinsic), on Behe-IDT and Dawkins-ET respectively; *p*s<.05 except as noted. However, none of the religiosity measures were influenced by MS, *t*(116) = 0.63, and *t*s(118) = 1.38 and 0.52, for the single-item scale, intrinsic, and extrinsic religiosity, respectively, all *ns*, suggesting that the effect of MS on views of Behe-IDT was not due to any change in religious belief. Indeed, the effect of MS on Behe-IDT held controlling for religiosity, *F*(1, 115) = 5.49 (based on the single-item measure of religiosity), and *F*(1, 116) = 7.19 (based on the intrinsic and extrinsic religiosity scales), *p*s<.05. Perhaps most important, religiosity did not moderate the effect of MS on Behe-IDT, *β*s = −.09, −.06, and −.06 for the single-item, intrinsic, and extrinsic religiosity scales, all *ns*; nor was there an interaction between religiosity and MS on Dawkins-ET, *β*s = −.11, −.04, and .01 for the three scales, all *ns* (these last analyses were conducted using multiple regression, rather than ANOVA, because the religiosity variable used in the interaction was continuous). In all studies, except where noted, *p* values for null findings on religiosity, religion, and Christianity as moderators were greater than .10; none of these were marginally significant.

We also tested whether type of religion moderated the effect of MS. Participants were classified as Christian (i.e., Christians, Christian-Orthodox, Catholic, and Protestant) or not Christian (i.e., Buddhist, Hindu, Jewish, Muslim, Sikh, Spiritual, and “None”). Christians were expected to be most supportive of IDT, given conceptual links between IDT and Creationism, so if IDT is a security-providing worldview that works in the same manner as religious ideologies, Christians should be most likely to embrace IDT in response to MS. Expected main effects of Christianity emerged on both Behe-IDT, *F*(1, 111) = 7.93, and Dawkins-ET, *F*(1, 111) = 7.54, *p*s<.05, indicating that Christians showed greater positivity than non-Christians toward Behe-IDT, and greater negativity toward Dawkins-ET. However, there was no interaction between Christianity and MS on either Behe-IDT or Dawkins-ET, *F*s(1, 111) = 0.21 and 1.17, both *ns*, indicating that MS did not have a stronger effect on these views among Christians. These results held when Catholics were classified as non-Christians rather than Christians, and this was the case in all studies.

Finally, MS had no effect on negative mood, *t*(119) = 1.43, *ns*; but slightly increased positive mood, *t*(119) = 2.00, *p*<.05, consistent with previous research [37]. Indeed, MS manipulations tend to have little impact on explicit affect, but effects emerge occasionally, as was the case in Studies 1 and 3 here. However, entering positive affect and negative affect as covariates in all five studies did not alter any results.

Thus, belief in IDT may, in part, be a normative response to heightened death awareness. However, the sample included in Study 1 was highly homogenous; it remains unclear whether students of more varied science backgrounds, better representing the educated public who support IDT, would respond similarly. Study 2 was designed to address this issue.

### Study 2

**Figure 2:**
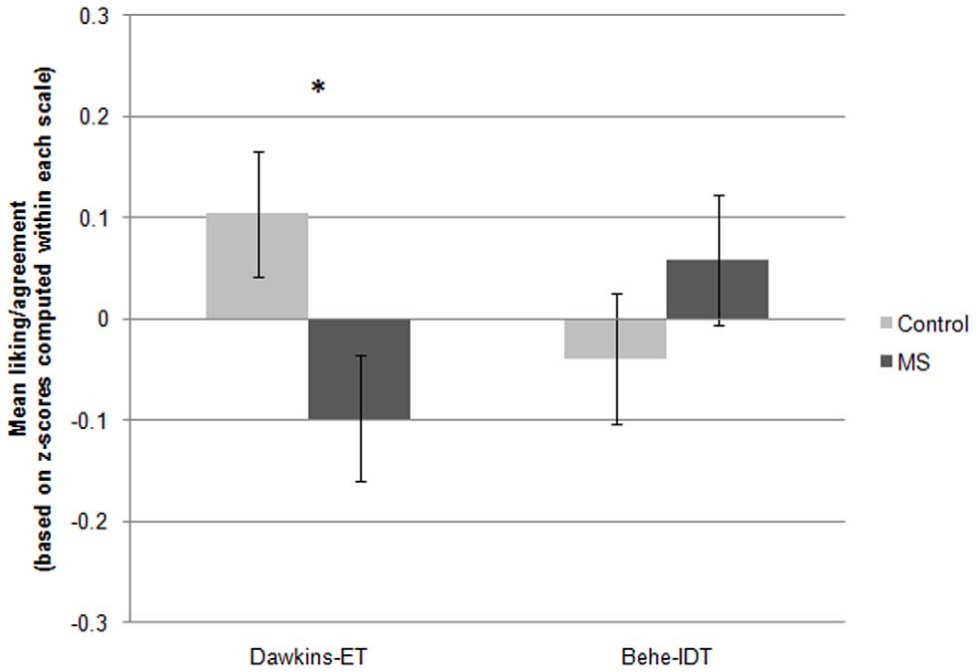
Effects of MS on liking of Behe and belief in IDT, and liking of Dawkins and belief in ET, Study 2. **Note**. Values are based on standard scores; means for each scale were computed by standardizing each of the six author-theory items that the scale comprised, around their common mean, and taking the mean of the resulting z-scores. Because the two scales were centered around different means, values on the two scales (Dawkins-ET and Behe-IDT) cannot be directly compared to each other. The overall interaction, which emerged from a mixed-measures analysis of variance (ANOVA), and the difference between the control and MS conditions on Dawkins-ET, based on a *t*-test, was significant, *p*<.05. Error bars represent the standard error of the mean. *p<.05.

A 2 (Behe-IDT vs. Dawkins-ET)×2 (MS vs. control) mixed-measures ANOVA on the author-theory scales revealed an interaction between the within-subjects factor of author-theory and the between-subjects factor of MS condition, *F*(1, 350) = 4.21, *p*<.05, indicating that MS led to opposite responses to the two authors and theories: relatively greater positivity toward Behe-IDT [though not significantly greater than control, *t*(350) = 1.07, *ns*], but significantly greater *negativity* toward Dawkins-ET, *t*(350) = 2.30, Cohen's *d* = .24, *p*<.05 (see Figure 2). The interaction and main effect on ET both held when using the two-item scale of views toward ET and IDT, *F*(1, 349) = 4.28, and *t*(350) = 2.09, both *p*s<.05, suggesting that these results are not due to MS affecting views of the authors without also affecting views of the relevant theories.

Religiosity again related positively to views of Behe-IDT, *r* = .39, and negatively to Dawkins-ET, *r* = −.34, both *p*s<.05. MS had no effect on religiosity, *t*(339) = 0.43, *ns*, and the effect of MS on Dawkins-ET held controlling for religiosity, *F*(1, 338) = 3.81, *p* = .05, as did the interaction, *F*(1, 338) = 2.54, *p* = .06. As in Study 1, there was no interaction between religiosity and MS on Dawkins-ET, *β* = −.01, nor on Behe-IDT, *β* = −.01, both *ns*. Again classifying participants as Christian or non-Christian, a two-way interaction emerged between Christianity and author-theory, *F*(1, 348) = 60.54, *p*<.05, indicating that Christians preferred Behe-IDT, *F*(1, 188) = 25.77, *p*<.05, but non-Christians preferred Dawkins-ET, *F*(1, 162) = 39.58, *p*<.05. However, Christianity did not moderate the interaction between MS and author-theory, *F*(1, 348) = 0.29, nor the effect of MS on Behe-IDT, *F*(1, 348) = 0.05, or Dawkins-ET, *F*(1, 348) = 1.38, all *ns*, indicating that decreased support for ET in response to MS was not driven by Christians.

In general, the findings of Study 2 replicate those of Study 1. Across studies, MS influenced participants' views of ET and IDT; in Study 1 this emerged as greater support for IDT, and in Study 2 as greater antagonism toward ET. This difference between studies may be due to a lower baseline belief in ET in the more diverse Study 2 sample, or to reluctance among Study 1's psychology student participants to question ET while participating in scientific research. Nonetheless, the studies converge to suggest that university educated individuals' views of IDT and ET can be influenced by existential threat.

However, both studies were restricted to student populations, leaving it unclear whether widespread support for IDT and skepticism toward ET seen among post-collegiate Americans can be attributed to terror management processes. Similarly, we do not know whether individuals who have not attended college would respond similarly. Individuals who do not subscribe to the scientific cultural worldview may be less likely to implicitly use their beliefs about scientific (or seemingly scientific) theories as a way of managing existential threat. Thus, to the extent that the results of Studies 1 and 2 were due to the scientific framing of IDT and ET, they may not generalize to less educated individuals.

In addition, to retain ecological validity, both Studies 1 and 2 manipulated ET and IDT using statements written by two prominent authors, thus examining how MS influences views of these theories as they are actually encountered by the average science student, teacher, or other well-read individual. However, as a consequence of this design, it remains possible that the differences found were due not to the relative merits of the theories, but rather to something unique about the writing of the two passages. Study 3 was designed to address these issues.

### Study 3

**Figure 3:**
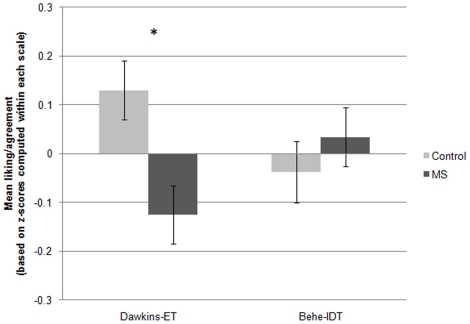
Effects of MS on liking of Behe and belief in IDT, and liking of Dawkins and belief in ET, Study 3. **Note.** Values are based on standard scores; means for each scale were computed by standardizing each of the six author-theory items that the scale comprised, around their common mean, and taking the mean of the resulting z-scores. Because the two scales were centered around different means, values on the two scales (Dawkins-ET and Behe-IDT) cannot be directly compared to each other. The overall interaction, which emerged from a between-subjects ANOVA, and the difference between the control and MS conditions, based on a *t*-test on Dawkins-ET, were significant, *p*<.05. Error bars represent the standard error of the mean. *p<.05.

A 2 (MS vs. control)×2 (Behe-IDT vs. Dawkins-ET) between-subjects ANOVA revealed an interaction between author-theory and MS, *F*(1, 828) = 7.71, *p*<.05, replicating Study 2 and indicating that MS led to opposite responses to the two theories: relatively greater positivity toward Behe-IDT [though not significantly different from control, *t*(402) = 0.81, *ns*], and significantly greater negativity toward Dawkins-ET, *t*(426) = 2.99, Cohen's *d* = .30, *p*<.05 (see Figure 3). The interaction and main effect on ET held using the two-item scales assessing views of the theories only, *F*(1, 828) = 4.86, and *t*(426) = 2.38, both *p*s<.05, again suggesting that results are not due to MS affecting views of the authors without also affecting views of the corresponding theories.

Religiosity again related positively to views of Behe-IDT and negatively to Dawkins-ET, *r*s = .21 and −.52, respectively, *p*s<.05. MS did not affect religiosity, *t*(828) = 0.72, *ns*, and the interaction between MS and author-theory held controlling for religiosity, *F*(1, 825) = 7.36, *p*<.05, as did the main effect on Dawkins-ET, *F*(1, 423) = 8.29, *p*<.05. Neither religiosity, *β* = .04, nor Christianity, *F*(1, 824) = 0.89, moderated the interaction, nor the main effect on Dawkins-ET, *β* = .01 for religiosity, and *F*(1, 424) = 0.25 for Christianity, all *ns*; there also were no interactions between these variables and MS on Behe-IDT, *β* = −.04, for religiosity, and *F*(1, 400) = 0.75 for Christianity, both *ns*.

To examine whether participants' educational background—a rough indicator of their subscription to the scientific worldview—moderated effects, we first converted education to a dichotomous variable based on a median split (college graduates vs. “some college” or less). There was no three-way interaction between education, MS, and author-theory, *F*(1, 821) = 0.13, nor was there an interaction using the full categorical education variable, *F*(5, 802) = 0.23, nor when treating education as continuous, *β = *.00, all *ns*. Education also did not moderate the effect of MS on Dawkins-ET, *F*(1, 421) = 0.03 (dichotomous variable), *F*(5, 412) = 0.29 (categorical), and *β = *.02 (continuous); nor did interactions emerge on Behe-IDT, *F*(1, 400) = 0.57 (dichotomous), *F*(6, 390) = 0.86 (categorical), and *β = *.02 (continuous); all *ns*. Controlling for education, the interaction between author-theory and MS held, *F*(1, 824) = 7.63, *p*<.05, as did the main effect of MS on Dawkins-ET, *F*(1, 422) = 8.75; *p*s<.05.

These findings suggest that the present results are not driven by individuals with a strong educational background, but rather seem to represent a terror management strategy used regardless of education. This is informative for the distinction that emerged between Studies 1 and 2, regarding whether participants were more likely to support IDT or disavow ET in response to MS. Given that the specific effects of Study 3 mirrored those of Study 2, and in both studies participants were *not* drawn from a population of psychology students participating in research for psychological course credit (as they were in Study 1), these findings support our interpretation of the difference between studies as related to individuals' longstanding beliefs about ET. Individuals who are not necessarily psychology students, may or may not have strong educational backgrounds, and tend to hold weaker pro-ET views than do psychology students, appear more willing to shift their views of ET in response to MS, compared with psychology students participating in psychological research as part of a course requirement. Supporting this interpretation, the highly diverse Study 3 sample showed greater control-condition positivity toward IDT and negativity toward ET than did the less diverse Study 1 and 2 samples; examining the four items that directly addressed views of IDT and ET (i.e., asking whether each is the “best explanation we have of species' origins” and a “solid theory supported by a great deal of evidence”), control condition pro-IDT and pro-ET *M*s in Study 3 = 2.97 and 3.11, compared to Study 1 *M*s = 2.38 and 3.68, between-samples *t*(252) = 3.73, and *t*(268) = 3.13, *p*s<.05; and compared to Study 2 *M*s = 2.57 and 3.70, between-samples *t*(365) = 5.04 and *t*(382) = 4.59, *p*s<.05 (Study 3 scores were transformed from a 7-point to a 5-point scale to make these comparisons).

Together, these studies suggest that individuals ranging in age from late adolescence to late adulthood, from a diverse range of socioeconomic, regional, and educational backgrounds, tend to respond to MS with increased support for IDT or decreased support for ET. Despite the superficial differences between these responses, at an underlying conceptual level they are coherent; participants respond to existential concerns by increasing their relative preference for an apparently “scientific” theory (i.e., IDT) that can provide a sense of meaning and purpose to the human endeavor, and/or decreasing their support for the theory that fails to do so.

However, the results thus far do not tell us whether these effects were, as we surmise, due to an activated search for greater meaning and purpose in response to existential threat. Given the consistent pattern of results—across studies, participants responded by rejecting the scientific theory suggesting that human life is meaningless and/or embraced the theory suggesting life is meaningful—this seems likely. However, few previous studies have established a motivational causal process (beyond avoidance of death-related anxiety) underlying the effects of MS. In one relevant study a motivation to increase feelings of control was found to account for MS effects [38], but that motive is unlikely to account for the present results because both ET and IDT depict humans as at the mercy of external forces (i.e., low in control). Thus, to increase our understanding of why views of IDT/ET are influenced by existential threat, Study 4 examined whether a search for greater meaning might account for effects.

In addition, one limitation of Studies 1–3 is that religious belief was assessed only during the experimental session, and stable attitudes about evolution could not be assessed separately from the dependent variable, so we had no baseline measure of religion or attitudes toward ET. Furthermore, the religious disposition that might be expected to most strongly influence views of IDT and ET, religious fundamentalism, was not assessed. Thus, Study 4 included a follow-up assessment, in which we measured religious (including fundamentalism) and evolution beliefs several months after the experiment, on a subset of the sample.

### Study 4

If participants in Studies 1–3 responded to MS by evidencing discomfort with ET, a scientific theory that may be taken to indicate the meaninglessness of human existence, or embracing IDT, a seemingly scientific theory that may be taken to indicate greater meaning in human life, *because* existential concerns promote the acceptance of seemingly scientific theories that provide such meaning and the rejection of those that do not, then framing ET as having the potential to provide meaning and purpose should remove or reverse these effects. Under such conditions, psychology students should not need to embrace IDT in the face of existential threat, as they did in Study 1, because the more normative theory associated with their scientific worldview would no longer be inconsistent with the need to find greater meaning.

We tested this account in Study 4 by assigning half the participants to read an excerpted passage by cosmologist and science writer Carl Sagan arguing that humans can attain meaning and purpose by seeking to understand the natural origins of life [35]. In this passage, Sagan explicitly states that even if humans are “merely matter,” we still can find purpose, but it must be one that we work out for ourselves. The passage articulates a way in which greater meaning can be found from embracing naturalism, so if the findings from Studies 1–3 were due to the apparent absence of such meaning in ET compared to IDT, reading this passage should weaken or reverse those effects.

#### Main study

**Figure 4:**
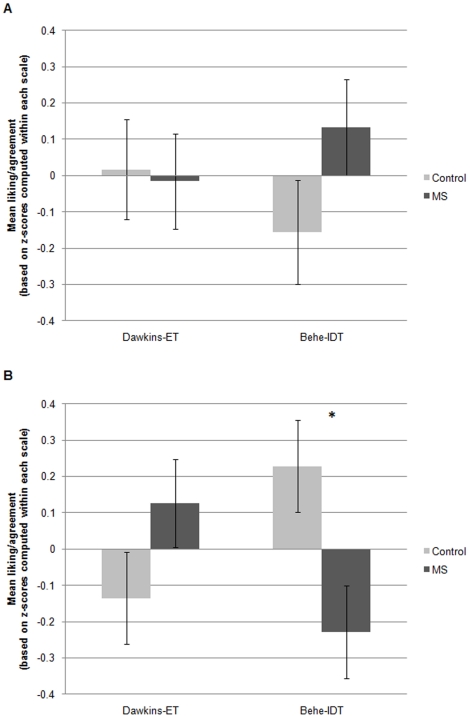
Effects of MS on liking of Behe and belief in IDT, and liking of Dawkins and belief in ET, for participants who did not read Sagan's excerpt about naturalism (Panel A) and those who did (Panel B), Study 4. **Note.** Values are based on standard scores; means for each scale were computed by standardizing each of the six author-theory items that the scale comprised, around their common mean, and taking the mean of the resulting z-scores. Because the two scales were centered around different means, values on the two scales (Dawkins-ET and Behe-IDT) cannot be directly compared to each other. The overall three-way interaction, based on a between-subjects ANOVA, and, in Panel B, the two-way interaction and the difference between the control and MS conditions, based on a between-subjects ANOVA and a *t*-test on Behe-IDT, respectively, were significant, *p*s<.05. Error bars represent the standard error of the mean. *p<.05.

A 2 (Sagan vs. no-Sagan)×2 (MS vs. control)×2 (Dawkins-ET vs. Behe-IDT) between-subjects ANOVA revealed the predicted three-way interaction, *F*(1, 257) = 6.96, *p*<.05 (see Figure 4); this interaction held when the two-item scales reflecting views of ET/IDT only were used instead, *F*(1, 257) = 6.07, *p*<.05. To interpret this three-way interaction, we conducted two 2 (MS vs. control)×2 (Dawkins-ET vs. Behe-IDT) between-subjects ANOVAs, separately for participants in the Sagan and no-Sagan conditions. In the no-Sagan condition, the two-way interaction was not significant, *F*(1, 126) = 1.26, *ns*, but, as in Study 1, participants trended toward increased positivity toward Behe-IDT in response to MS, though here the difference from control did not reach significance, *t*(61) = 1.38, *p* = .17. In contrast, in the Sagan condition, a significant two-way interaction, *F*(1, 131) = 7.44, *p*<.05, revealed the reverse pattern: when participants read Sagan's passage suggesting that naturalism can be a source of meaning, they responded to MS with significantly *decreased* positivity toward Behe-IDT, *t*(64) = 2.58, Cohen's *d* = .64, *p*<.05, and relatively increased positivity toward Dawkins-ET, though not significantly different from control, *t*(67) = 1.45, *p* = .15. This represents a full reversal of the effect found in Study 1, where MS led to significantly increased positivity toward Behe-IDT compared to control. Both the MS×author interaction, and the main effect on Behe-IDT, in the Sagan condition, held when using the two-item scales reflecting views of ET and IDT only, *F*(1, 131) = 2.69, and *t*(64) = 2.86, *p*<.05.

Religiosity was again correlated with Behe-IDT and Dawkins-ET, *r*s = .19 and −.30, both *p*s<.05, and a two-way interaction between Christianity and author-theory, *F*(1, 249) = 20.76, *p*<.05, indicated that Christians showed no preference between Behe-IDT and Dawkins-ET, whereas non-Christians tended to prefer Dawkins-ET [*t*(98) = 1.01, *ns*, for Christians; and *t*(163) = 8.63, for non-Christians, *p*<.05]. MS did not affect religiosity, *t*(260) = 1.05, *ns*, and the three-way interaction between Sagan condition, MS, and author-theory held controlling for religiosity, *F*(1, 253) = 7.12, as did the interaction between MS and author-theory in the Sagan condition, *F*(1, 128) = 8.39, and the main effect on Behe-IDT in this condition, *F*(1, 62) = 6.96; *p*s<.05. Neither religiosity, *β* = −.06, nor Christianity, *F*(1, 249) = 0.04, moderated the three-way interaction, nor the two-way interaction in the Sagan condition, *β* = .04 for religiosity and *F*(1, 127) = 0.02 for Christianity; all *ns*. Religiosity also did not moderate the main effect of MS on Behe-IDT in the Sagan condition, *β* = .10, *ns*, but there was a marginal Christianity×MS interaction on Behe-IDT in this condition, *F*(1, 62) = 3.39, *p* = .07, indicating that non-Christians who read Sagan became more negative toward Behe-IDT in response to MS than did Christians, although effects were in the predicted direction for both groups (*M*s = −0.59 vs. 0.01 for non-Christians and −0.28 vs. −0.21 for Christians, in MS and control conditions, respectively). There was no interaction between religiosity and MS on Dawkins-ET, *β*s = .14 and .20 in the Sagan and no-Sagan conditions, nor between Christianity and MS on Dawkins-ET in either condition, *F*(1, 65) = 1.69 and *F*(1, 63) = 0.20; all *ns*.

#### Follow-up study

The three-way interaction between MS, Sagan/no Sagan, and author-theory emerged in this sub-sample, *F*(1, 92) = 8.98, and held controlling for scores on all four measures of stable religious views, *F*(1, 83) = 8.32, and acceptance of evolution, *F*(1, 91) = 8.74; *p*s<.05. As in the full sample, a two-way interaction between MS and author-theory emerged in the Sagan condition, *F*(1, 47) = 6.20, *p*<.05. There was also an interaction in the no-Sagan condition, *F*(1, 45) = 3.18, *p*<.05 one-tailed. In both no-Sagan and Sagan conditions, simple effects emerged on views of Behe-IDT, in opposite directions, such that MS increased positivity in the no-Sagan condition, replicating Study 1, and decreased positivity in the Sagan condition, *t*s(24) = 2.09 and 2.24, Cohen's *d*s = .88 and .91, respectively, *p*s<.05. Both effects held controlling for scores on all four religion measures and ET acceptance, *F*(1, 19) = 7.24, in the Sagan, and *F*(1, 17) = 5.60, in no-Sagan condition; *p*s<.05. As in the main study, the three-way and two-way interaction in the Sagan condition, and the main effect on IDT in the Sagan condition, all held when the two-item scales assessing views of ET/IDT only were used instead; *F*(1, 92) = 6.44, *p*<.05; *F*(1, 47) = 4.05, *p* = .05; *t*(24) = 2.14, *p*<.05.

Finally, with only one exception, none of the four measures of stable religious views or ET acceptance moderated any of the interactions or main effects in this subsample. The one exception was an extrinsic religiosity×MS interaction on Behe-IDT in the no-Sagan condition, *β = *.54, *p*<.05, indicating that the effect of MS on Behe-IDT was weaker among individuals high in extrinsic religiosity. Given that similar effects did not emerge with any of the other religion measures, and this particular effect is not easily interpretable, it is unlikely to be reliable. In sum, the present results cannot be attributed to stable individual differences in religious belief, fundamentalism, or views of evolution, and do not vary depending on these beliefs or views.

#### Summary and limitations

One potential limitation of this study is that we did not include a neutral control passage in the no-Sagan condition, given that even a seemingly neutral passage might have elicited unexpected priming effects. This resulted in an approximately 2-minute additional delay following the MS manipulation in the Sagan condition. However, this delay-length difference is unlikely to account for effects, because: (a) in Studies 1 and 2, participants read counterbalanced passages by Dawkins *and* Behe, and completed each measure immediately after each passage, yet no order effects emerged despite the varying delay lengths; and (b) if the additional delay influenced results, previous research suggests that it would either increase the effect, if the delay heightened participants' terror-management response, or decrease the effect, if the delay allowed terror-management processes to wane [39]. There is no indication, conceptual or empirical, that an additional delay would completely *reverse* effects. Thus, it is considerably more likely that the strong differences found between the Sagan and no-Sagan conditions resulted from the substantive content of the Sagan manipulation.

The findings of Study 4 converge with those of Studies 1, 2, and 3, but add to our conceptual understanding. Specifically, the finding that reading Sagan's excerpt moderated the interaction between MS and attitudes toward ET versus IDT suggests that a desire to see human life as having greater meaning and purpose likely underlies our previous effects. Reading the Sagan passage apparently dissuaded participants from embracing IDT as a way of managing existential concerns, and in fact made participants facing existential threat more antagonistic toward IDT, presumably because it threatened the theory that is the true mainstay of their scientific worldview and that could now be seen as providing existential meaning. This result is important because it addresses the process underlying the causal link between MS and scientific beliefs.

Given these findings, certain individuals who are more deeply invested in the scientific worldview (e.g., scientists) may embrace ET in response to existential threat even without reading about how naturalism can be meaningful. Although we found no evidence of moderation by educational background in Study 3, very few participants in that study worked in scientific fields (only 15% reported working in technical or health related fields), so even if such individuals responded differently, their responses would be unlikely to produce a significant interaction. Thus, in Study 5 we directly sampled natural-science students. For these individuals, ET is not simply a theory they have learned in some courses, it is the cornerstone of their academic life, and an identity-defining worldview. Thus, we expected that these participants would not reject ET in the face of existential threat, but would instead more staunchly support the theory, given that, like Sagan, they may view naturalism as providing human life with meaning and purpose.

### Study 5

**Figure 5:**
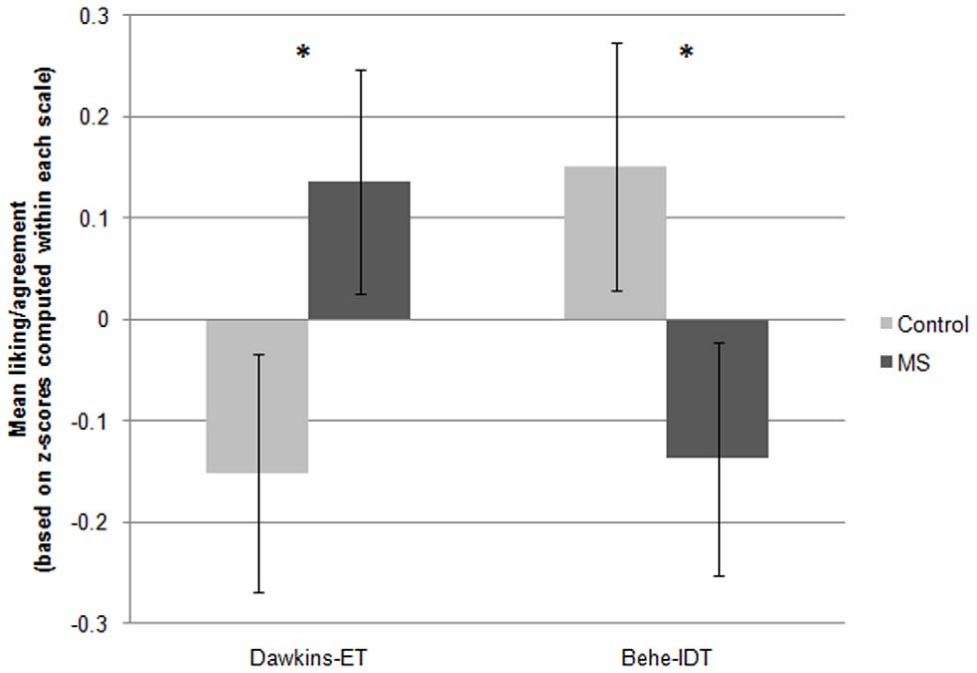
Effects of MS on liking of Behe and belief in IDT, and liking of Dawkins and belief in ET, in a sample of natural science students, Study 5. ***Note*** **.** Values are based on standard scores; means for each scale were computed by standardizing each of the six author-theory items that the scale comprised, around their common mean, and taking the mean of the resulting z-scores. Because the two scales were centered around different means, values on the two scales (Dawkins-ET and Behe-IDT) cannot be directly compared to each other. The overall interaction, based on a mixed-measures ANOVA, and the main effects on Dawkins-ET and Behe-IDT, based on *t*-tests, were significant, *p*s<.05 one-tailed. Error bars represent the standard error of the mean. *p<.05 one-tailed.

A 2 (Behe-IDT vs. Dawkins-ET)×2 (MS vs. control) mixed-measures ANOVA revealed an interaction between the within-subjects factor of author-theory and the between-subjects factor of MS, *F*(1, 94) = 4.19, *p*<.05, indicating that, in contrast to the diverse samples of participants in Studies 1–4, natural-science students trended toward greater *negativity* toward Behe-IDT in response to MS, *t*(96) = 1.76, Cohen's *d* = .35, *p*<.05 one-tailed, but greater *positivity* toward Dawkins-ET, *t*(94) = 1.80, Cohen's *d* = .33, *p*<.05 one-tailed (see Figure 5). This interaction held when we included only natural-science majors, *F*(1,75) = 3.93, *p* = .05, rather than the full sample which included majors and non-majors, but all students who reported taking at least 2 university-level natural science courses. The interaction also held when using the two-item measure of views of ET/IDT only, *F*(1, 94) = 5.17, and interestingly, both main effects, on IDT and ET, were significant using these scales, *t*s (94) = 3.23 for IDT and 1.47 for ET, all *p*s<.05.

Religiosity again correlated positively with Behe-IDT, and negatively with Dawkins-ET, *rs* = .32 and −.34, *p*s<.05. MS had no effect on religiosity, *F*(1, 94) = .00, *ns*, and both the interaction and simple effect on Behe-IDT held controlling for religiosity, *F*(1, 92) = 4.45, *p*<.05, and *F*(1, 93) = 2.95, *p*<.05 one-tailed. These effects also held controlling for the number of natural-science courses taken, *F*(1, 93) = 4.99, *p*<.05, and *F*(1, 95) = 3.90, *p* = .05. Neither religiosity nor number of natural-science courses moderated the main effect of MS on Behe-IDT, *β*s = .02 and −.16; both *ns*. These variables also did not interact with MS to produce an effect on Dawkins-ET, *β*s = .17 and −.04, both *ns*. Christianity did not moderate the interaction, *F*(1, 81) = .01, nor the effect of MS on Behe-IDT, *F*(1, 83) = 0.72, both *ns*. There was no Christianity×MS interaction on Dawkins-ET, *F*(1, 81) = 1.25, *ns*.

Thus, Study 5 suggests that there is at least one group of individuals who do not embrace IDT or reject ET in response to MS: individuals invested in natural-science research. Here, heightened existential threat led to the opposite response from that seen among psychology students in Studies 1 and 4, the diverse students in Study 2, and diverse adults in Study 3. The present responses were, however, similar to those of the psychology students in Study 4 who learned that naturalism can be a source of greater meaning (i.e., those in the Sagan condition). Together, Studies 4 and 5 thus suggest that individuals who can find greater meaning in a naturalist perspective respond to existential threat by rejecting IDT and trending toward greater belief in ET. Presumably, shifting these views in response to MS allows these students to enhance symbolic immortality by reaffirming the scientific perspective that is a major part of their worldview *and* provides meaning and purpose. These findings thus support our account of the causal process underlying the effects found in Studies 1–3, and delineate an important boundary condition for these effects.

### General Discussion

The present findings demonstrate that reminders of one's mortality—inducing a state of mortality salience—promote relative support for IDT, and skepticism toward ET. Individuals respond to existential threat by becoming more accepting of a theory that offers a greater sense of meaning by depicting human life as having ultimate purpose (while appearing consistent with the scientific worldview), and/or less supportive of the theory that is the true mainstay of the scientific worldview but seems to offer little in the way of existential comfort. These findings also suggest that a desire to find greater meaning in human life accounts for this effect (at least the effect of mortality salience on belief in IDT), because it is reversed by making ET more meaningful, and among natural-science students for whom ET is presumably already meaningful. The findings are notable because they (a) help explain why some people are motivated to believe in IDT and doubt ET in terms of fundamental psychological drives; (b) account for the underlying causal process; and (c) emerged regardless of preexisting religious ideologies, religious affiliation, or (with one highly limited exception, discussed below) views of evolution. This last point suggests that although religion influences baseline beliefs in IDT and ET, it cannot account for the impact of MS on these views. Given previous research suggesting that many MS effects are heightened, or occur only, among individuals with certain preexisting belief systems or cultural associations, the fact that we found no moderators of MS effects—other than the extent to which naturalism is seen as meaningful—suggests that embracing IDT or rejecting ET may be a unique, broadly appealing mechanism that addresses the existential concerns of religious and, for the most part, more scientifically oriented individuals alike. In contrast, explicitly religious ideologies tend to be fairly parochial, limiting their appeal and making them viable defenses only for those who already believe in a supernatural god [15].

Yet, an exception emerged in Study 5, where individuals whose life goals require strong acceptance of ET showed the opposite responses. Like those explicitly taught, in Study 4, to view naturalism as a source of meaning, natural-science students responded to MS with stronger antagonism toward IDT. This provides converging support for the causal process found in Study 4, and suggests that rejecting IDT can be a source of existential comfort for a limited population of individuals. These individuals are not simply those steeped in the scientific cultural worldview—presumably psychology undergraduates fall into that category—but rather those who more specifically view evolution as a critical part of their understanding of the world and a source of meaning and purpose.

### Specificity of the Effect

Although the precise direction of the effect—whether it emerged more strongly as antagonism toward ET or support for IDT—differed across studies, this was likely due to sampling differences. The same pattern of results was observed in the samples that, demographically, most resembled each other—those in Studies 1 and 4, and Studies 2 and 3—the former of which revealed a greater effect on Behe-IDT, and the latter on Dawkins-ET. Study 1 and 4 participants were largely middle-to-upper class and well-versed (if not firmly entrenched) in ET and the scientific cultural worldview. These individuals appeared to be largely unmovable in their views of ET, probably because the theory has become such a mainstay of their worldview as social-science students that, even if they would like to reject it when confronted with existential threat, this desire is negated by a compulsion to affirm ET as an important worldview component. Instead, these individuals modified their more malleable attitudes—those related to IDT, a theory with which they are almost certainly less familiar.

In contrast, Study 2 and 3 participants were drawn from a broader community; most were recruited by a survey company and either attended a wide variety of universities across North America (Study 2) or were adults whose age and socioeconomic status represented almost the entire spectrum of the U.S. population (Study 3). Although these individuals are likely to be at least nominally familiar with ET, the theory is unlikely to be as important to their understanding of the world as it is for psychology undergraduates. This may explain why they were willing to espouse more negativity toward ET in order to defend against MS. It is unclear why these individuals would not also show stronger support for IDT in such conditions, but given trends in that direction, it may be that rejecting ET is simply the more powerful means of coping with existential threat, at least for individuals who do not feel a sense of loyalty to the theory. Regardless, it is noteworthy that while demographic factors may influence the specific nature of this response, they do not change whether the response occurs; as was shown in the Study 4 follow-up, effects held controlling for stable views of ET.

More broadly, the fact that a consistent pattern emerged across studies, but with differences in the specific nature of the pattern depending on sample characteristics, suggests that embracing IDT and rejecting ET may be functionally similar in terms of regulating the potential for existential anxiety. Indeed, in the studies where participants completed both scales (Studies 1, 2, and 5) the two scales were always negatively correlated, with *r*s ranging from −.21 to −.47, all *p*s<.05. In practice, it is not particularly important whether individuals respond to MS by increasing support of IDT or decreasing support of ET, because IDT proponents tend to argue for both the merits of IDT and the limitations of ET [40]. Nonetheless, although we strongly suspect that differences across studies were due to sampling, further research is needed.

### Causal Process

The present research addresses critical questions about the process underlying effects found. Thus far, studies have demonstrated that the effects of mortality salience may be mediated by death-thought accessibility [23] and the potential for anxiety [41], but there is little evidence regarding the specific motivational nature of different terror management mechanisms; that is, few studies have directly examined *why* particular worldviews assuage death-related anxiety, by testing whether responses are moderated by the extent to which they resolve some need or motive. Here, we expected that IDT would be more appealing than ET because it better addresses a motivation to find meaning and purpose in the face of existential threat; findings from Studies 4 and 5 support this account. Although Study 5's natural-science student participants may have been driven by a more general desire to embrace an already accepted worldview or reject a theory antagonistic to it [42], the fact that they were able to do so, given the results of the previous four studies, suggests that they also view ET as a source of expansive meaning. However, future studies are needed to probe the specific causal process underlying *these* individuals' responses, to determine whether it is the same as that of the psychology students in Study 4 who read the Sagan passage.

The results of Studies 4 and 5 also may have implications for the process underlying other effects of mortality salience, such as gravitation toward religious ideas [15]. They also pinpoint the problem with ET for individuals seeking security in the face of existential threat. ET is typically presented as the highly materialist and utilitarian process that evolution is; as Dawkins explains, “unordered atoms… group themselves into ever more complex patterns until they end up manufacturing people.” Only when individuals are also told, “If there's nothing in here but atoms, does that make us less, or does that make matter more?”—implying that naturalism can reveal purpose in human life—do individuals reject IDT in response to heightened MS. Future studies are needed to examine whether manipulations along these lines, demonstrating the potential for meaningfulness in the natural sciences, generalize beyond psychology students who may already be motivated to find such meaning in science.

### Implications and Conclusions

These findings have implications for our understanding of how existential concerns influence views of scientific theories and individuals' willingness to accept them, and for the success of the IDT movement. No previous study has examined whether psychological motives influence the ongoing debate between proponents of IDT and ET—a debate of great importance to the future of science and science education. The present research suggests that attitudes toward scientific (or seemingly scientific) views and ideologies can be partly shaped by unconscious psychological motives to maintain security and ward off existential angst through the cultivation of meaning and purpose.

In addition to providing a psychological explanation for the popularity of IDT and antipathy toward ET, the present findings challenge the conventional assumption that attitudes toward such scientifically framed theories are determined solely by factors such as logic, educational background, and ideology, though previous research suggests that such factors clearly play a role [43]–[47]. This is consistent with other recent studies on the motivational underpinnings of social cognition, which have shown that core insecurities regularly influence overt attitudes about ostensibly unrelated sociopolitical issues, and that such beliefs are thus often not objective, rationally derived constructions, but, rather, influenced by fundamental motivations such as the need to protect the self against psychological insecurity (e.g., existential, epistemic, personal, or relational uncertainty; [7], [48]–[53]). The present research builds on and extends these previous findings by showing that such processes generalize to attitudes and beliefs in the scientific domain.

In sum, although religious ideology plays a large role in public support for IDT and antagonism toward ET, these attitudes, held by both religious and non-religious individuals, can be partly explained by IDT's potential for assuaging existential anxiety, and ET's apparent lack of an existentially compelling solution to life's origins.

## Supporting Information

Text S1 Passages Used as Stimuli in Studies 1, 2, 4, and 5.
(DOC)

Text S2 Supplementary Analyses.
(DOC)

Text S3 Passages Used as Stimuli in Study 3.
(DOC)

Text S4 Passage Used as Stimulus in Study 4.
(DOC)
