# Supplementary material for: Death and Science: The Existential Underpinnings of Belief in Intelligent Design and Discomfort with Evolution
Source: PLoS One. 2011 Mar 30;6(3):e17349. doi: 10.1371/journal.pone.0017349 (PMC3068159; doi:10.1371/journal.pone.0017349)
Supplement: Text S1 — Passages Used as Stimuli in Studies 1, 2, 4, and 5. (DOC) [file pone.0017349.s001.doc]

**Text S1**

**Passages Used as Stimuli in Studies 1, 2, 4, and 5**

# ***Dawkins-Evolutionary Theory passage:***

# Darwin's theory of evolution by natural selection is satisfying because it shows how simplicity could change into complexity, how unordered atoms could group themselves into ever more complex patterns until they ended up manufacturing people. Darwin provides a solution, the only feasible one so far suggested, to the deep problem of our existence. . . Today the theory of evolution is about as much open to doubt as the theory that the earth goes round the sun. Many evolutionary transitions are elegantly documented by more or less continuous series of gradually changing intermediate fossils. Darwinian evolution shatters the illusion of design within the domain of biology, and teaches us to be suspicious of any kind of design hypothesis in physics and cosmology as well. The full implications of Darwin's revolution have yet to be widely realized. Darwinism encompasses all of life—human, animal, plant, and bacterial. Darwinian evolution, as one reviewer has observed, is the most important natural truth that science has yet discovered. No serious biologist doubts the fact that evolution has happened.

# All excerpts reproduced in this supplementary file remain the copyright of the original copyright holders.

# ***Behe-Intelligent Design Theory passage:***

# Darwinian evolution is being pushed to its limits by discoveries in biochemistry. Over the last fifty years, discoveries in biology, physics, astronomy, and cosmology, suggest that life and the universe manifest signs of real design. Further, many evolutionary biologists have acknowledged fundamental problems with Darwinian evolution as an explanation for the complexity and apparent design of living organisms. As a result of both these developments, many scientists and philosophers now think that the universe and life appear designed because they really were. These scientists advocate an alternative theory of biology and cosmological origins known as the theory of intelligent design, or, simply, design theory. Design theorists believe that scientific evidence actually points to intelligent design. This result, of cumulative efforts to investigate life at the molecular level, is so unambiguous that it must be ranked as one of the greatest achievements in the history of science. The observation of the intelligent design of life is as momentous as the observation that the earth goes around the sun or that disease is caused by bacteria.

# All excerpts reproduced in this supplementary file remain the copyright of the original copyright holders.
