# Supplementary material for: Death and Science: The Existential Underpinnings of Belief in Intelligent Design and Discomfort with Evolution
Source: PLoS One. 2011 Mar 30;6(3):e17349. doi: 10.1371/journal.pone.0017349 (PMC3068159; doi:10.1371/journal.pone.0017349)
Supplement: Text S2 — Supplementary Analyses. (DOC) [file pone.0017349.s002.doc]

**Text S2**

**Supplementary Analyses**

Because standard scores were used in all five studies, means on the two scales (Dawkins-ET and Behe-IDT) cannot be directly compared to each other (both are 0 when standard scores are used). However, to ensure that actual mean responses on these scales were not at floor or ceiling, we also computed scores for each participant by summing across the 6 items on each scale. In no study was the overall mean of these summed scores near ceiling or floor; the possible range in Studies 1, 2, 3, and 5 was 6-46, and in Study 4 it was 6-42; and in Study 1 the means were 32.94 and 25.73 for Dawkins-ET and Behe-IDT, respectively; in Study 2 these values were 31.06 and 26.27, respectively; in Study 3, means were 26.70 and 28.11, respectively; in Study 4, means were 28.80 and 25.57; and in Study 5, means were 33.68 and 25.83. In all five studies these means differed significantly from each other, Study 1 *t*(239) = 8.12, Study 2 *t*(702)= 6.96, Study 3 *t*(830) = 1.97, Study 4 *t*(263) = 7.33, and Study 5 *t*(194) = 6.89, all *p*s < .05, indicating that participants in Studies 1, 2, 4, and 5 (all university students) tended to prefer Dawkins-ET over Behe-IDT, whereas participants in Study 3 (the diverse non-student sample) tended to prefer Behe-IDT over Dawkins-ET.
