# Supplementary material for: Death and Science: The Existential Underpinnings of Belief in Intelligent Design and Discomfort with Evolution
Source: PLoS One. 2011 Mar 30;6(3):e17349. doi: 10.1371/journal.pone.0017349 (PMC3068159; doi:10.1371/journal.pone.0017349)
Supplement: Text S3 — Passages Used as Stimuli in Study 3. (DOC) [file pone.0017349.s003.doc]

**Text S3**

**Passages Used as Stimuli in Study 3**

***Evolutionary Theory passage:***

Although the universe and life appear designed, many scientists and philosophers now agree that they really weren’t. These evolutionary theorists believe that scientific evidence clearly points to evolution. This result is so unambiguous that it must be ranked as one of the greatest achievements in the history of science. Darwin's theory of evolution by natural selection is satisfying because it shows how simplicity could change into complexity, how unordered atoms could group themselves into ever more complex patterns until they ended up manufacturing people. Darwin provides a solution, the only feasible one so far suggested, to the deep problem of our existence. The full implications of Darwin's revolution have yet to be widely realized. Darwinism encompasses all of life—human, animal, plant, and bacterial. Darwinian evolution is the most important natural truth that science has yet discovered. The observation of the evolution of life is as momentous as the observation that the earth goes around the sun or that disease is caused by bacteria.

# All excerpts reproduced in this supplementary file remain the copyright of the original copyright holders.

***Intelligent Design passage:***

People often feel like the universe and life must have been designed, and many scientists and philosophers now agree that this is because they really were. These design theorists believe that scientific evidence clearly points to intelligent design. This result is so unambiguous that it must be ranked as one of the greatest achievements in the history of science. Intelligent design theory is satisfying because it shows how complexity exists, how atoms could group themselves into ever more complex patterns until they form people. Intelligent design provides a solution, the only feasible one so far suggested, to the deep problem of our existence. The full implications of the intelligent design revolution have yet to be widely realized. Intelligent design encompasses all of life—human, animal, plant, and bacterial. Intelligent design is the most important truth that science has yet discovered. The observation of the intelligent design of life is as momentous as the observation that the earth goes around the sun or that disease is caused by bacteria.

# All excerpts reproduced in this supplementary file remain the copyright of the original copyright holders.
