# Supplementary material for: Death and Science: The Existential Underpinnings of Belief in Intelligent Design and Discomfort with Evolution
Source: PLoS One. 2011 Mar 30;6(3):e17349. doi: 10.1371/journal.pone.0017349 (PMC3068159; doi:10.1371/journal.pone.0017349)
Supplement: Text S4 — Passage Used as Stimulus in Study 4. (DOC) [file pone.0017349.s004.doc]

**Text S4**

**Passage Used as Stimulus in Study 4**

***Sagan passage*:**

It is very reasonable for humans to want to understand something of our context in a broader universe, awesome and vast. It is also reasonable for us to want to understand something about ourselves. And understanding the nature of the world and the nature of ourselves is, to a very major degree, I believe, what the human enterprise is about. Truth should be pursued, and science helps us pursue it; science gives us meaning. All we have to do is maintain some tolerance for ambiguity, because right now science does not have all the answers. This tolerance goes with the courageous intent to greet the universe as it really is, not to foist our emotional predispositions on it but to courageously accept what our explorations and knowledge tell us. The more likely we are to assume that the solution comes from something outside science, the less likely we are to solve our problems ourselves. If we are merely matter that is intricately assembled, is this really demeaning? If there's nothing in here but atoms, does that make us less, or does that make matter more? We make our purpose. And we have to work out what that is, for ourselves.

# All excerpts reproduced in this supplementary file remain the copyright of the original copyright holders.
